# Automatic Segmentation of Eight Tissue Classes in Neonatal Brain MRI

**DOI:** 10.1371/journal.pone.0081895

**Published:** 2013-12-17

**Authors:** Petronella Anbeek, Ivana Išgum, Britt J. M. van Kooij, Christian P. Mol, Karina J. Kersbergen, Floris Groenendaal, Max A. Viergever, Linda S. de Vries, Manon J. N. L. Benders

**Affiliations:** 1 Department of Neonatology, Wilhelmina Children’s Hospital/University Medical Center Utrecht, Utrecht, The Netherlands; 2 Image Sciences Institute, University Medical Center Utrecht, Utrecht, The Netherlands; Centre Hospitalier Universitaire Vaudois Lausanne - CHUV, UNIL, Switzerland

## Abstract

**Purpose:**

Volumetric measurements of neonatal brain tissues may be used as a biomarker for later neurodevelopmental outcome. We propose an automatic method for probabilistic brain segmentation in neonatal MRIs.

**Materials and Methods:**

In an IRB-approved study axial T1- and T2-weighted MR images were acquired at term-equivalent age for a preterm cohort of 108 neonates. A method for automatic probabilistic segmentation of the images into eight cerebral tissue classes was developed: cortical and central grey matter, unmyelinated and myelinated white matter, cerebrospinal fluid in the ventricles and in the extra cerebral space, brainstem and cerebellum. Segmentation is based on supervised pixel classification using intensity values and spatial positions of the image voxels. The method was trained and evaluated using leave-one-out experiments on seven images, for which an expert had set a reference standard manually. Subsequently, the method was applied to the remaining 101 scans, and the resulting segmentations were evaluated visually by three experts. Finally, volumes of the eight segmented tissue classes were determined for each patient.

**Results:**

The Dice similarity coefficients of the segmented tissue classes, except myelinated white matter, ranged from 0.75 to 0.92. Myelinated white matter was difficult to segment and the achieved Dice coefficient was 0.47. Visual analysis of the results demonstrated accurate segmentations of the eight tissue classes. The probabilistic segmentation method produced volumes that compared favorably with the reference standard.

**Conclusion:**

The proposed method provides accurate segmentation of neonatal brain MR images into all given tissue classes, except myelinated white matter. This is the one of the first methods that distinguishes cerebrospinal fluid in the ventricles from cerebrospinal fluid in the extracerebral space. This method might be helpful in predicting neurodevelopmental outcome and useful for evaluating neuroprotective clinical trials in neonates.

## Introduction

Cerebral volumetric segmentation and voxel-based morphometry have been applied to MR images of newborn infants, and have shown to be of great additional value in studying brain development at early stages [1-3]. In neonates, various risk factors such as preterm birth, chronic lung disease or intra-uterine growth restriction may influence and alter brain tissue volumes [4-6] . Several studies have illustrated the correlation between brain volumes and neurodevelopmental outcome in childhood and adolescence of prematurely born subjects [1,7,8] However, data regarding brain volumes at term-equivalent age (TEA) and long-term neurodevelopmental outcome are rather limited [9].

To study subtle differences in volumes of brain tissues in large cohorts, an automatic segmentation method is mandatory since manual segmentations are very time-consuming and subjective. To assess brain development and maturation in newborns, it is necessary to identify different tissue classes. In the last decade, several semi-automatic [10-12] or automatic [13-19] neonatal brain segmentation algorithms have been described. Some segmentation methods distinguished unmyelinated white matter (UWM) from myelinated white matter (MWM) [15-17], and/or central grey matter (CeGM) from cortical grey matter (CoGM) [11,13,16,17]. While the cerebellum (CB) was shown to be very important in the neurodevelopment in preterm infants [20], only a few methods have segmented it separately [11,17-19,21]. Furthermore, pronounced or increased ventricles are known to be related to cerebral atrophy and adverse outcome [22]. Segmentation of the ventricles separately from CSF was only performed by a few other groups [10,18,21]. However, some only excluded the cerebrospinal fluid in the extracerebral space [10,19], although the interhemispheric subarachnoid space is described to be increased and of prognostic value in preterm infants. Recently, Gousias et al. [21] designed a protocol for manual annotation of neonatal brain into 50 regions. Using this protocol, the authors compared total and regional brain volumes in preterm- and term-born infants scanned around TEA and found that there are only small regional differences between the groups. However, these conclusions were made based on a relatively small dataset.

Automatic methods developed for segmentation of adult brain with MRI are generally not applicable for segmentation in neonatal scans. In neonatal brain images the contrast between different tissue types is lower compared with the contrast in adult scans because the majority of white matter in neonates is unmyelinated and has higher water content. In addition, the scan time in neonatal brain imaging is limited because of possible motion of the infants, which reduces the signal-to-noise ratio [6]. 

We propose an automatic method for segmentation of eight brain structures in neonatal MRI. The method utilizes a supervised pixel segmentation approach. Each brain voxel is described by a set of intensity and spatial features based on which a supervised pixel classification is performed. The method segments UWM, MWM, CeGM, CoGM, cerebrospinal fluid both in the extracerebral space (referred to as ‘CSF’) and in the ventricles (VENT), brainstem (BS) and CB. The method is based on an earlier publication by our group [13]. Both methods perform segmentation of brain tissue classes using supervised voxel classification utilizing intensity and spatial information. Here proposed method contains two major novelties compared to the previous work. First, an average brain image is introduced which is used for extraction of spatial features. Second, in addition to earlier presented segmentation of CoGM, CeGM, total white matter, and cerebrospinal fluid, this method also performs segmentation of cerebellum, ventricles separately from CSF, UWM and MWM separately, and brainstem.

## Materials and Methods

### Patients and MR images

The study was approved by the Medical Ethics Committee of our institute and written informed parental consent was obtained for all infants. This MRI study was performed in preterm infants around TEA (n=108) [23,24], with a gestational age at birth <31 weeks, from January 2007 until June 2008. Seven infants with MRI without cerebral pathology, without movement artifacts and with a normal outcome at two years of age (BSID-III; mean MDI score 109 ± 17, mean PDI score 105 ± 7) were chosen for manual segmentation. Patients’ characteristics are shown in Table 1. 

**Table 1:** Patient characteristics presented separately for the large cohort of 101 infants and for the seven infants with reference brain annotations.

**Variable**	**Total cohort (n=101)**	**Manual annotated scans (n=7)**
Gender (male/female)	56/45	1/6
Gestational age (weeks)	28.4[25.1-30.9]	28.9[25.6-30.9]
Birth weight (grams)	1129[630-1910]	1118[650-1705]
Postmenstrual age at scan (weeks)	41.7[39.6-43.6]	41.3[40.6-42.1]
Bronchopulmonary dysplasia (n)	44 (43.6%)	3 (42.9%)
Patent Ductus Arteriosus (n)	35 (35.7%)	1 (14.3%)
Culture proven sepsis (n)	49 (48.5%)	3 (42.9%)
Woodward white matter score [38]	8[5-12]	8[5-10]
BSITD-III total motor composite score, corrected age	107[73-142]	105[97-118]
BSITD-III cognitive composite score, corrected age	103[80-140]	109[95-145]

Table lists mean value and range is given in brackets (mean[range]), there were no significant differences between the two groups. BSITD (Bayley scale of infant and toddler development, third edition [37]).

 For each infant, axial 3DT1-weighted (TR=9.4 ms; TE=4.6 ms; scan time=3.44 min, FOV=180x180; scan matrix=512x512; consecutive sections with thickness=2.0 mm; number of sections=50) and axial T2-weighted images (TR=6293 ms; TE=120 ms; scan time=5.40 min; FOV=180x180; scan matrix=512x512; consecutive sections with thickness=2.0 mm; number of sections=50) were acquired on a 3.0 Tesla MR system (Philips Healthcare, Best, The Netherlands) using a sense head coil. The infants were sedated with 50-60 mg/kg chloralhydrate by gastric tube 15 minutes prior to the examination. During MR examination, the infants were placed in a vacuum fixation pillow to reduce movement and hearing protection was administered while heart rate, transcutaneous oxygen saturation and respiratory rate were monitored. A neonatologist was present throughout the procedure.

### Manual segmentation

In the seven infants, chosen for manual segmentation, the T2-weighted scans were manually annotated slice by slice by one of the authors (BJMvK). Each brain voxel was assigned to one of the eight tissue classes (CoGM, CeGM, UWM, MWM, CSF, VENT, BS and CB) by mouse painting. The labeling was indicated by a color overlay: each tissue type was represented by one color. When labeling MWM, both T1- and T2-weighted images were used to precisely define the tissue border. We used T1 images for MWM segmentation since early changes of myelination are best seen on these images in term equivalent infants. In the manual segmentation process each voxel was assigned to one tissue type only. The manual segmentation of one image-set of one patient took approximately 100 hours. These manual segmentations were verified independently by three neonatologists with at least five years of experience in reading neonatal MRI scans, corrected according to their findings and reevaluated in a consensus meeting. The manual segmentations were considered as reference standard for training and validation of the segmentation method

To estimate inter-observer agreement, a subset of five out of seven scans was selected for manual annotations by other observers. In these scans, each of the eight tissue types was segmented in three slices, determined for each tissue separately. The selected slices were those numbered as 25^th^ percentile, median and 75^th^ percentile of the slices visualizing each given tissue. The manual annotations were performed following the aforementioned protocol by five medical students trained specifically for this task performed them. None of them has been involved in the annotation of the reference standard. Segmentations were finally checked and corrected by expert neonatologists.

### Automatic segmentation algorithm

The proposed segmentation algorithm is based on supervised pixel classification [25]. Each voxel was described with intensity and spatial features. Based on these features, each brain voxel was assigned to one of the eight tissue classes using K-nearest neighbor classifier. 

Before classification was employed, several preprocessing steps were carried out. First, to compensate for acquisition inhomogeneity effects, a shading correction was performed to the T1- and T2-weighted MR images. This was implemented following the algorithm by Likar et al. [26]. 

(Next, intra- subject registration was carried out. Considering that acquisition takes under 10 minutes, in spite of sedation, occasionally subjects move during scanning. To enable exploitation of intensity characteristics from both T1- and T2-weighted scans, these scans needed to be aligned. Given that MR imaging results in the distortion of tissue in different sequences, the scans were registered using affine and subsequently elastic intra-patient registration. This was modeled by B-splines at three resolutions (a Gaussian pyramid with a sub sampling factor of two in each direction) to avoid local minima in the cost function. As a cost function negative mutual information was used. For the optimization of the cost function an iterative stochastic gradient descent optimizer was employed. At each resolution 200 iterations of the stochastic gradient descent optimizer were performed. The derivative of the mutual information was calculated with 5000 image samples, randomly chosen at every iteration. 32 histogram bins were used. The affine and elastic registrations were performed using elastiX (http://elastix.isi.uu.nl) [27].

Finally, to utilize spatial features, scans of all patients needed to be transformed into a common coordinate system. For this purpose, an average T2-weighted brain image was constructed. This was achieved by iterative registration and summation of the T2-weighted images of all patients in the cohort. Thus, initial average was obtained by summation of the T2-weighted scans. Subsequently, all images were aligned with this initial average by registration. This way transformed images were again averaged and this image represented the current average brain. This process was repeated iteratively until the stable average brain image was obtained, as determined by visual inspection. This way bias towards a single reference image was removed from the average brain image. To achieve a coarse alignment, affine registration was performed with the same parameters described above, except here 16 histogram bins were used when computing the derivative of the cost function. Subsequently, the alignment was refined using elastic registration modeled by B-splines. Again multi-resolution strategy with three resolutions was used. Final grid spacing was 16 voxels. 16, 32 and 32 histogram bins were used, respectively. The remaining parameters were the same as listed above. 

 Each voxel was described by the features to perform classification, i.e. to assign each voxel in the image to one of the given tissue classes. T1- and T2-weighted images provided intensity information, and voxel position i.e. x-, y- and z- coordinates in the coordinate system of the average brain gave spatial characteristics. For that purpose, the T2-weighted scan of each patient was elastically registered to the average brain using affine and elastic registration. The registration was performed using the same parameters described above.

To account for different ranges of features, all features were scaled to zero mean and unit variance prior to classification [25]. Pilot experiments showed that the best results were obtained using a k-nearest-neighbor classifier (kNN) with k set to 50 in the former method [13,28]. The classifier assigned each voxel a posterior probability for each tissue type and background. This way, probabilistic segmentation was generated for each tissue.

Finally, to obtain brain segmentation in the original T2-weighted scan, probabilistic segmentation result was transferred from the space of the average brain to the patient’s T2-weighted image coordinate system by inverse registration transformation. To obtain binary segmentations, each voxel was assigned to the tissue class with the highest posterior probability determined by the using kNN classifier [25]. 

Note that due to different sizes of brain tissue classes, images and consequently training data, consisted of unequal number of voxels, thus training samples, per brain tissue class. It has been shown that in an unbalanced data set, i.e. data set in which minority class is represented by small number of samples and majority class by large portion of all samples, the classification performance often decreases [29]. To improve the performance different sampling strategies have been proposed [30-32]. We have performed local correction by inspecting neighborhood of each sample in the feature space proposed by Tan [30]. This approach assigns larger weight to neighbors of small classes and little weight to neighbors from large classes. This way correction for differences in the *a priori* probabilities of different classes in the neighborhood of a given sample is performed. The weight *w*_*i*_ **of the class *i* is determined using the following formula:

$$w_{i}=\frac{1}{Num(C_{i})/Min_{j=1,...,N}{\left(Num(C_{j}^{d})\right)}^{\frac{1}{exponent}}}$$

where *Num* denotes the cardinality, and *C*_*i*_  neighbors of class *i* in the neighborhood of the observed sample among N nearest neighbors. *exponent* is a parameter >1.

Given the weight *w*_*i*_, the corrected posterior probability of a voxel for each class *i* was determined by multiplying the posterior probability of the voxel for class *i* determined by the kNN classifier by the corresponding class weight *w*_*i*_.

### Experiments

To perform segmentation seven patients with a manually set reference standard were used. Evaluation was performed in a ‘leave-one-out’ fashion. This means that each patient brain was classified using the training set composed from the images of the remaining six patients. This was performed seven times, so each scan was once a test scan. Owing to the large number of samples, i.e. voxels in each brain, 20% of brain voxels were randomly selected from each training image. The selected samples were merged into one set and used for training the classifier. This reduced computation time and computer memory usage.

 To determine whether the correction for the unbalanced data is needed in this task, segmentation was determined by assigning each sample class label with the largest a posteriori probability determined by the classification without correction (i.e. using posterior probabilities determined by standard kNN classification) and with correction (i.e. using the above described weights to obtain corrected posterior probabilities). When applying correction, several values between 1 and 75 were evaluated to find the *exponent* giving the best performance.

 After the best classification strategy was determined, automatic results were compared with the manual segmentations to quantitatively evaluate the performance of the method. Since the voxels in the manually set reference standard were assigned to a single tissue type, binary segmentations were compared with the reference standard using the Dice similarity coefficient (DSC) determined as follows:

$$DSC=\frac{2\times TP}{2\times TP+FP+FN}$$

where TP was the number of true positive, TN number of true negative, FP number of false positive, and FN number of false negative voxels.

In addition, the sensitivity ($\frac{TP}{TP+FN}$), and specificity ($\frac{TN}{TN+FP}$) of the segmentation were calculated.

Subsequently, volumes of the different brain tissues were calculated in two ways: 1) directly from the probability maps, and 2) using the binary majority class segmentations. For both methods, the volume per tissue class was compared with the manual segmentations.

To establish whether the proposed method is robust on a large set of images, it was subsequently applied to the remaining 101 infants of the cohort. The training set was in this case created using all seven images with manual reference annotations. As in leave-one-out experiments, 20% of the brain voxels were randomly selected from each of the seven images and subsequently merged into the training set. 

Considering that the reference standard was not available in these scans, the probabilistic segmentation results were evaluated by visual inspection of expert observers. Three observers performed the analysis and each scan was presented to only one of them, The visual inspection of all images was performed slice by slice on the *complete* scan using mainly T2-weighted scans for 7 structures, and both T1- and T2-weighted image when evaluating MWM segmentation. The segmentation result of each tissue was graded on a scale from 1 to 5 (1=very poor, 2=poor, 3=moderate, 4=good, 5=very good). The observers were allowed to assign values from 1 to 5 with 0.5 increments.

In addition, automatic segmentations of the seven scans with reference annotations were also evaluated visually. Results of the visual assessment were related to the quantitative evaluation.

## Results

**Figure 1:**
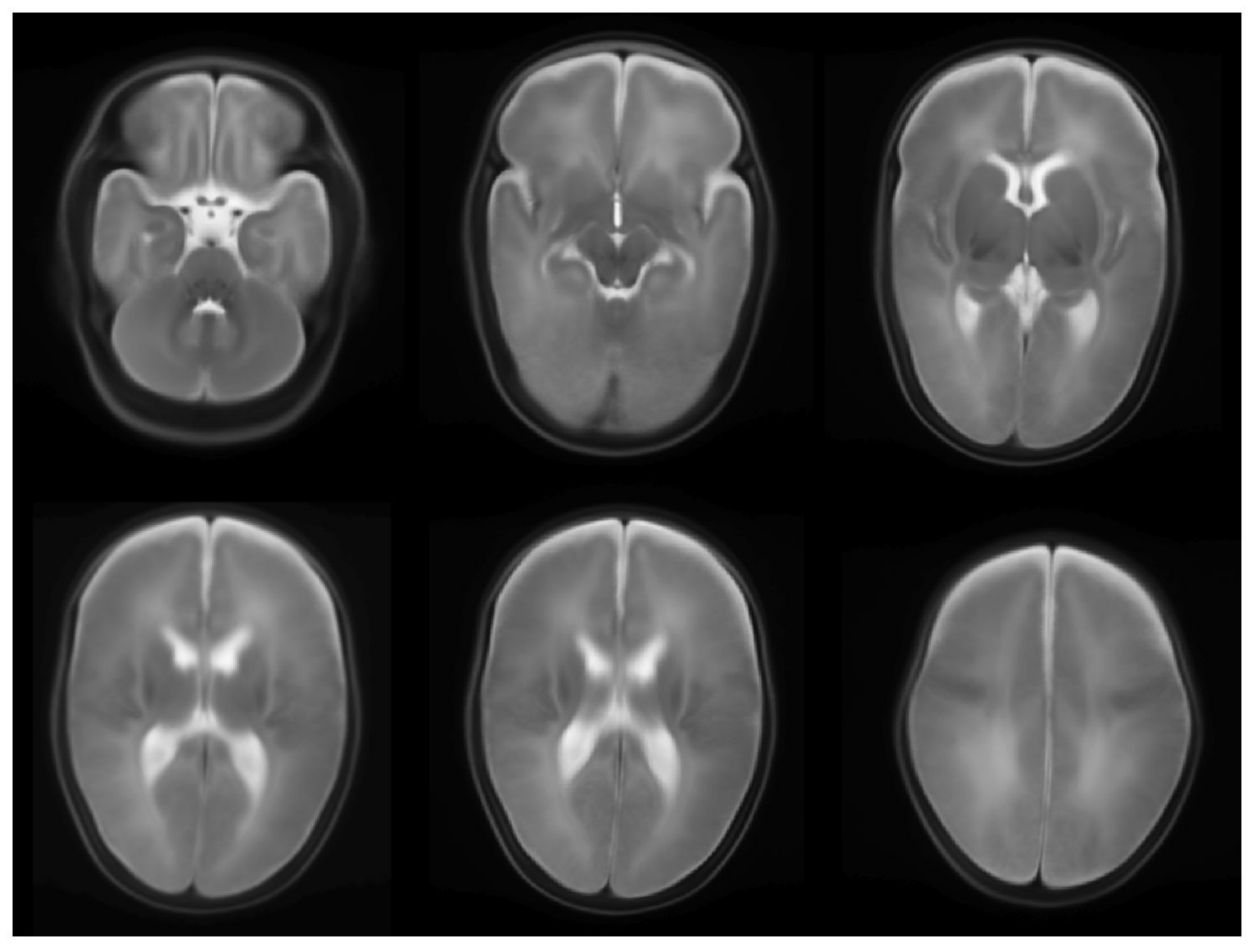
Several slices showing average brain image. This was achieved by iterative registration and summation of the T2-weighted images of all patients in the cohort.

Figure 1 shows sections from the average brain image. As illustrated in the figure, major brain structures can be clearly identified. Visual inspection of the alignment between test T2-weighted scan and the average brain showed good alignment. 

In the segmentation, optimal classification strategy was first determined. In comparison to the segmentation without correction for differences in the prior probabilities for different tissue classes, increasing the *exponent* in the correction strategy, resulted in larger probabilities for tissues with a small percentage of samples in the data set at the expense of lower probabilities for the segmentation of tissues represented by many samples. This was most pronounced in the segmentation result of the myelinated white matter and cerebellum. Nevertheless, weighting did not influence the binary segmentation result. When increasing the *exponent* (i.e. when its value was getting closer to the maximal tested value), visual inspection of the segmentation results showed that the segmentations were becoming more similar to those obtained without the correction. The same trend was observed by computation of the resulting tissue volumes. Because this correction has not led to the improvement in the segmentation performance, results without the correction were used for further analysis. 

**Figure 2:**
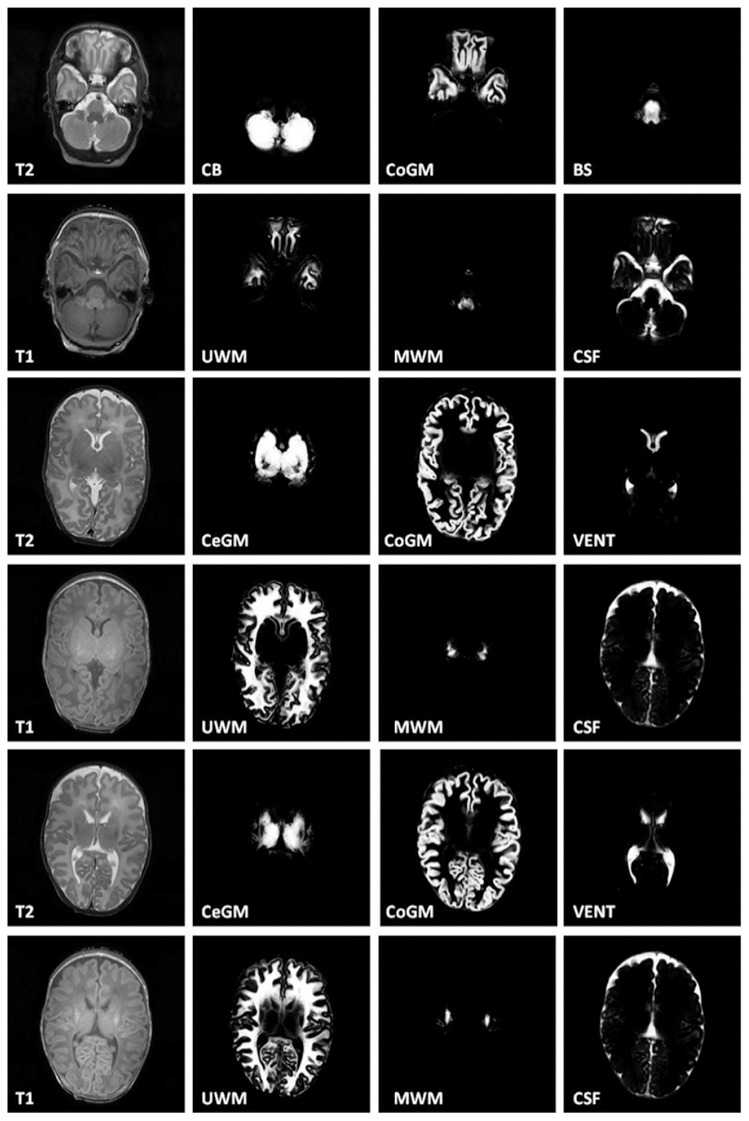
Probabilistic segmentations of intracranial tissues on several levels. Abbreviations: T2: T2-weighted image; CB: Cerebellum; CoGM: Cortical gray matter; BS: Brainstem; T1: T1-weighted image; UWM: Unmyelinated white matter; MWM: myelinated white matter; CSF: Cerebrospinal fluid.

Next, automatically obtained binary segmentation was compared to the reference standard. Note again that the binary segmentation was obtained from the probabilistic results using majority class segmentation. Table 2 lists the results in terms of DSC, sensitivity and specificity. Figure 2 shows probabilistic segmentations at three levels in the brain in one randomly selected infant. Performance of the second observer segmentation was evaluated using the same metric and the results are listed in Table 3. Results indicate that for most tissues agreement between an observer and the reference standard is similar to the agreement between the automatic segmentation and the reference standard. Exceptions are CoGM and CSF. Retrospective analysis revealed that the annotations of the second observer mostly differ from the reference annotations along cortical surface, especially in sulci where CSF is poorly visible and grey matter forms closed shape. 

**Table 2:** Binary segmentation result evaluated in terms of Dice overlap measure, sensitivity and specificity averaged over seven patients.

Tissue type	DSC	Sensitivity	Specificity
Cerebellum	0.919 (0.008)	0.924	0.999
Myelinated white matter	0.470 (0.125)	0.422	1.000
Central grey matter	0.911 (0.012)	0.918	0.995
Ventricles	0.838 (0.035)	0.828	0.997
Unmyelinated white matter	0.854 (0.021)	0.870	0.923
Brainstem	0.838 (0.024)	0.833	0.998
Cortical grey matter	0.827 (0.030)	0.870	0.894
Cerebrospinal fluid	0.751 (0.071)	0.727	0.930

Standard deviations are given in brackets. The binary segmentations were obtained from the probabilistic segmentations using majority class voting.

**Table 3:** Binary segmentation result performed by the second observer evaluated in terms of Dice overlap measure, sensitivity and specificity averaged over seven patients.

Tissue type	DSC	Sensitivity	Specificity
Cerebellum	0.935(0.006)	0.905	0.999
Myelinated white matter	0.465 (0.127)	0.409	0.999
Central grey matter	0.939 (0.010)	0.928	0.999
Ventricles	0.880 (0.028)	0.898	0.999
Unmyelinated white matter	0.895 (0.337)	0.953	0.986
Brainstem	0.843 (0.636)	0.830	0.999
Cortical grey matter	0.759 (0.062)	0.650	0.996
Cerebrospinal fluid	0.687 (0.062)	0.908	0.977

Here evaluated manual annotations were performed for each tissue in three slices in a subset of five images. Standard deviations are given in brackets.

After the segmentation was evaluated, volumes of the segmented brain tissues were computed. Table 4 lists brain tissue volumes calculated from the manually set reference standard and automatically obtained probabilistic and binary segmentations. Average results over all seven patients are presented. No significant differences with the reference standard were observed for tissue volumes calculated using probabilistic segmentation results. However, the volumes calculated by binary majority class segmentation differ significantly from the reference values for CB and CoGM.

**Table 4:** Brain tissue volumes (in cc) averaged over seven patients with reference standard calculated by two different approaches: 1) probabilistic segmentation and 2) binary segmentation obtained using majority class.

**Tissue type**	**Reference standard**	**Probability map**	**Majority**
**Cerebellum**	28.6	28.9	32.6*
**Myelinated white matter**	1.7	2.0	1.3
**Central gray matter**	22.6	22.7	23.3
**Ventricles**	8.3	8.7	8.5
**Unmyelinated white matter**	164.7	171.0	173.0
**Brainstem**	6.3	6.1	6.4
**Cortical gray matter**	152.2	152.6	174.4*
**Cerebrospinal fluid**	92.2	82.2	99.0

The automatically obtained volumes were compared with the reference volumes. Statistically significant different volumes are marked with a *.

* : p-value < 0.01 paired samples t-test, with bonferroni correction

Finally, visual inspection of the automatic segmentation in the remaining cohort was performed. Results are listed in Table 5. Note that manually set reference standard for those 101 scans was not available, because manual segmentations are extremely time-consuming, and therefore, quantitative evaluation was not feasible. The results show that the method is robust. No automatic segmentation has been assigned grade 1 which would have indicated a very poor result. Minimum grade of 2 was assigned in segmentation of Vent, CoGM, and CeGM to only one scan, and twice in segmentation of BS. This automatic segmentation of the ventricles was poor likely due to substantial motion artifacts in the images. Boundaries of these ventricles are blurry and automatic segmentation overestimated their volume. Segmentation of CoGM followed tissue well, but posterior probabilities were often low where the CoGM was clearly present (see Figure 2: 3^rd^ row, 3^rd^ column). 

**Table 5:** Evaluation of automatic segmentation in 101 scans performed by visual assessment.

	**Images without reference**			**Images with reference**		
**Tissue type**	**Median**	**P25**	**P75**	**Median**	**P25**	**P75**
**Cerebellum**	4	4	4.5	4.5	4.25	5
**Myelinated white matter**	4	3	4.5	5	4	5
**Central gray matter**	4	4	5	5	4.75	5
**Ventricles**	4.5	4	5	5	4.75	5
**Unmyelinated white matter**	4	4	4.5	5	4.5	5
**Brainstem**	4	4	4.5	5	4.75	5
**Cortical gray matter**	4	3.5	4	5	4.25	5
**Cerebrospinal fluid**	4	3.5	4.5	5	4.25	5

Segmentation of each tissue was graded on scale from 1 to 5 (1=very poor, 2=poor, 3=moderate, 4=good, 5=very good). Evaluation is presented separately for images without the reference annotations and for seven images with the reference annotations.

P25, 25^th^ percentile; P75, 75^th^ percentile

Minimum grade of 2.5 was assigned to one case in segmentation of CSF where the tissue was automatically segmented out of the brain, probably due to inaccurately generated brain mask. Segmentations of CB and UWM were graded with minimal grade 4 assigned to six and ten scans, respectively. Most of the segmentations were assigned grades between 4 and 5, showing good overall performance in a large set. 

Visual evaluation of the automatic segmentations was also performed in seven scans with manual annotations. The results are also listed in Table 5. They show that higher scores were obtained in this subset of seven selected images than in the large set of 101 scans. This is likely because these seven images were selected so that no cerebral pathology was visible and no movement artefacts were present. These infants had normal outcome at two years of age. Therefore, the scans were likely easier for the automatic segmentation. However, relating these grades to the quantitative evaluation is difficult. Quantitative evaluation of the MWM segmentation revealed low DC, while visual assessment had a median score of 5. 

## Discussion

This study presents an automatic brain segmentation and quantification of eight cerebral tissue classes in neonatal MRI scans acquired at TEA. The method segmented CSF, ventricles and cerebellum, separately. The generated automatic segmentations in a large set of 108 infant scans were accurate according to expert visual inspection.

Several methods for (semi-)automatic brain segmentation in neonatal MRI scans have been presented earlier [10-19,21]. These algorithms were based on pattern recognition, image registration, mathematical morphology, or a combination. The method that is proposed here is an extension of an earlier publication by our group [13] describing a method for automatic segmentation of neonatal MRI scans which is also based on supervised pixel classification using intensity and spatial information. There are two major novelties of the method described in this work. First, an average brain image was introduced which was used for extraction of spatial features. Second, segmentation of cerebellum, ventricles separately from CSF, UWM and MWM separately, and brainstem was performed next to the segmentation of central and cortical grey matter, total white matter, and cerebrospinal fluid. 

The presented method distinguishes VENT from CSF. This distinction is important in preterm infants, as an increased amount of CSF due to a large subarachnoid space could be an indicator of e.g. brain atrophy [22]. An increase in total CSF or solitary increased ventricular volumes at TEA is associated with an impaired outcome in preterm infants [9,22]. Furthermore, in previous publications segmentation of the cerebellum has often been omitted. Besides the method presented here, manual and automatic cerebellar segmentation have been described [10.11,17,19,21]. The CB volume has shown to be very important in especially the cognitive development of the preterm infant [20]. Segmentation of the CB is a difficult issue, since this structure shows inhomogeneous signal intensities on both T1- and T2-weighted scans, varying from values similar to UWM to intensities of GM. The use of spatial information in the proposed method was helpful. However, separation of occipital CoGM and cerebellum remained difficult. A similar problem was observed in the segmentation of CoGM and CeGM. These tissues have similar intensities and can only be distinguished by spatial information and shape. Misclassifications were observed as oversegmentation of CB and CeGM, where some CoGM voxels were detected as CB or CeGM. Nevertheless, the achieved overlap measures (DSCs) of CB, CoGM and CeGM segmentations were high.

Considering that the previously published methods were applied to different sets of images acquired using different parameters (image resolution, magnetic field strength, scan orientation), and that they segmented different tissue classes, it is difficult to directly compare the segmentation results. In addition, in different studies validations were performed in a single, or only in several sections [15-17]. In the present study, evaluation of all eight structures was performed slice by slice on the *complete* scan. Because manual annotations of all eight tissue types in all image sections are extremely time consuming (about 100 hours/scan), the manual reference standard was set in a subset of seven scans. Quantitative evaluation could only be performed in these images, and the remaining segmentations in 101 scans were checked by visual inspection. 

Although it is difficult to compare, the obtained average DSC values (0.71 - 0.92, except 0.47 for MWM) are similar to those reported earlier (0.56-0.75 by Prastawa et al. [15], 0.72-0.92 by Weisenfeld and Warfield [16]^16^, 0.75-0.90 by Gui et al. [17], and 0.84-0.88 by Yu et al. [11]). We obtained the lowest DSCs for MWM. The segmentation of MWM in the preterm brain around TEA is a challenge. The maturation of the preterm brain is an ongoing process showing different degrees of myelination over a short period of time and it is difficult to define the exact ending of MWM and the beginning of CeGM. Additionally, the volume of MWM in the preterm infant at TEA is relatively small. This implies that a small difference in segmented volume compared with the reference volume resulted in a low DSC. It is questionable whether T1- and T2-weighted scans provide sufficient information for the segmentation of MWM. Since MWM is related to the volumetric analysis of fiber tracts in the developing brain, it may be better to determine MWM volume using diffusion tensor imaging [33].

The presented method showed some segmentation inaccuracies. First, a classification error was seen in UWM areas where the signal intensity approached that of CSF. A diffuse high signal intensity in UWM is a common finding in preterm infants at TEA [34,35]. In those infants, UWM was misclassified as CSF. This issue was also described by Yu et al. [11]. To correct for this, the authors performed a manual correction as the final step in their segmentation process. Second, misclassification error was observed at the border between CSF and CoGM. Owing to partial volume effects, the signal intensities of these border voxels were similar to UWM. The method used by Xue et al. [14] corrected voxels belonging to CSF that were misclassified as WM using expectation-maximization algorithm. Gui et al. [17] set anatomical conditions on voxel neighborhood to correct misclassification as UWM at the interface between CoGM and CSF. Weisenfeld and Warfield [16] corrected for partial volume effect by iterative relaxation labeling. Considering that the goal of our segmentation method is determination of brain tissue volumes, and the fact that these inaccuracies were negligible in volumetric measurements, to this end we have not performed any correction. However, future work will focus on improving these misclassifications as they would be important in reconstruction of the cortical surface.

From the obtained segmentations, the volume of each tissue type was calculated using probabilistic segmentation results and binary segmentation obtained using majority class voting. Considering that no statistically significant differences were found between the volumes calculated using the probabilistic and binary segmentation results, and that in addition, probabilistic segmentations were visually more appealing, we consider probabilistic segmentation the method of choice for obtaining volumetric measurements.

Execution times of segmentation algorithms were often not reported. The method of Weisenfeld and Warfield [16] reported run-time of about 120 min. Yu et al. [11] reported execution time of two hours per infant. The entire segmentation process of our method took about 20 minutes per infant on a standard PC.

Our method has limitations. We described an algorithm that results in a probabilistic segmentation of brain tissue types. However, we evaluated our method using manually set binary reference standard. Setting the probabilistic reference standard manually would be theoretically possible. An observer could have been asked to assign each voxel a probability that it belongs to a certain tissue type. Alternatively, a number of observers could have been asked to delineate a given tissue type, and averaging their segmentations could have served as an approximation of a manually set probabilistic segmentation [36]. However, considering work load this would require from a human expert, obtaining the probabilistic manually set segmentation was not feasible. Therefore, we have quantitatively evaluated binary segmentation result obtained from the probabilistic segmentation, and thereafter visually inspected both segmentations. 

 To conclude, this neonatal automatic brain segmentation method allows to distinguish cerebrospinal fluid from ventricles, as well as to segment cortical and central grey matter, (un)myelinated white matter, cerebellum and brainstem. Distinguishing these tissue classes is of great clinical value. Evaluation in a large set of patients indicates its applicability in clinical settings. 

## References

[1] Peterson BS, Anderson AW, Ehrenkranz R, Staib LH, Tageldin M et al. (2003) Regional brain volumes and their later neurodevelopmental correlates in term and preterm infants. Pediatrics 5: 939–948. PubMed: 12728069. 10.1542/peds.111.5.939 12728069

[2] Ment LR, Hirtz D, Hüppi PS (2009) Imaging biomarkers of outcome in the developing preterm brain. Lancet Neurol 8: 1042–1055. doi:10.1016/S1474-4422(09)70257-1. PubMed: 19800293. 19800293

[3] Zacharia A, Zimine S, Lovblad KO, Warfield S, Thoeny H et al. (2006) Early assessment of brain maturation by MR imaging segmentation in neonates and premature infants. AJNR Am J Neuroradiol 27: 972–977. PubMed: 16687526. 16687526 PMC7975748

[4] Tolsa CB, Zimine S, Warfield SK, Freschi M, Sancho Rossignol A et al. (2004) Early alteration of structural and functional brain development in premature infants born with intrauterine growth restriction. Pediatr Res 56: 132–138. doi:10.1203/01.PDR.0000128983.54614.7E. PubMed: 15128927. 15128927

[5] Thompson DK, Warfield SK, Carlin JB, Pavlovic M, Wang HX et al. (2007) Perinatal risk factors altering regional brain structure in the preterm infant. Brain 130: 667–677. PubMed: 17008333. 17008333 10.1093/brain/awl277

[6] Mewes AUJ, Hüppi PS, Als H, Rybicki FJ, Inder TE et al. (2006) Regional brain development in serial magnetic resonance imaging of low-risk preterm infants. Pediatrics 118: 23–33. doi:10.1542/peds.2005-2675. PubMed: 16818545. 16818545

[7] Martinussen M, Flanders DW, Fischl B, Busa E, Løhaugen GC et al. (2009) Segmental brain volumes and cognitive and perceptual correlates in 15-year-old adolescents with low birth weight. The Journal of pediatrics 155, 848–853.e1 10.1016/j.jpeds.2009.06.015 PMC5875423 19683725

[8] Nosarti C, Giouroukou E, Healy E, Rifkin L, Walshe M et al. (2008) Grey and white matter distribution in very preterm adolescents mediates neurodevelopmental outcome. Brain 131: 205–217. PubMed: 18056158. 18056158 10.1093/brain/awm282

[9] Inder TE, Warfield SK, Wang H, Hüppi PS, Volpe JJ (2005) Abnormal cerebral structure is present at term in premature infants. Pediatrics 115: 286–294. doi:10.1542/peds.2004-0326. PubMed: 15687434. 15687434

[10] Nishida M, Makris N, Kennedy DN, Vangel M, Fischl B et al. (2006) Detailed semiautomated MRI based morphometry of the neonatal brain: preliminary results. NeuroImage 32: 1041–1049. doi:10.1016/j.neuroimage.2006.05.020. PubMed: 16857388. 16857388

[11] Yu X, Zhang Y, Lasky RE, Datta S, Parikh NA et al. (2010) Comprehensive brain MRI segmentation in high risk preterm newborns. PLOS ONE 5: e13874. doi:10.1371/journal.pone.0013874. PubMed: 21079730. 21079730 PMC2975631

[12] Warfield SK, Kaus M, Jolesz FA, Kikinis R (2000) Adaptive, template moderated, spatially varying statistical classification. Med Image Anal 4: 43–55. doi:10.1016/S1361-8415(00)00003-7. PubMed: 10972320. 10972320

[13] Anbeek P, Vincken KL, Groenendaal F, Koeman A, van Osch MJ et al. (2008) Probabilistic brain tissue segmentation in neonatal magnetic resonance imaging. Pediatr Res 63: 158–163. doi:10.1203/PDR.0b013e31815ed071. PubMed: 18091357. 18091357

[14] Xue H, Srinivasan L, Jiang S, Rutherford M, Edwards AD et al. (2007) Automatic segmentation and reconstruction of the cortex from neonatal MRI. NeuroImage 38: 461–477. doi:10.1016/j.neuroimage.2007.07.030. PubMed: 17888685. 17888685

[15] Prastawa M, Gilmore JH, Lin W, Gerig G (2005) Automatic segmentation of MR images of the developing newborn brain. Med Image Anal 9: 457–466. doi:10.1016/j.media.2005.05.007. PubMed: 16019252. 16019252

[16] Weisenfeld NI, Warfield SK (2009) Automatic segmentation of newborn brain MRI. NeuroImage 47: 564–572. doi:10.1016/j.neuroimage.2009.04.068. PubMed: 19409502. 19409502 PMC2945911

[17] Gui L, Lisowski R, Faundez T, Hüppi PS, Lazeyras F et al. (2012) Morphology-driven automatic segmentation of MR images of the neonatal brain. Med Image Anal 16: 1565–1579. doi:10.1016/j.media.2012.07.006. PubMed: 22921305. 22921305

[18] Cardoso MJ, Melbourne A, Kendall GS, Modat M, Robertson NJ et al. (2013) AdaPT: An adaptive preterm segmentation algorithm for neonatal brain MRI. NeuroImage 65: 97–108. doi:10.1016/j.neuroimage.2012.08.009. PubMed: 22906793. 22906793

[19] Kuklisova-Murgasova M, Aljabar P, Srinivasan L, Counsell SJ, Doria V et al. (2011) A dynamic 4D probabilistic atlas of the developing brain. NeuroImage 54: 2750–2763. doi:10.1016/j.neuroimage.2010.10.019. PubMed: 20969966. 20969966

[20] Allin M, Matsumoto H, Santhouse AM, Nosarti C, AlAsady MH et al. (2001) Cognitive and motor function and the size of the cerebellum in adolescents born very pre-term. Brain 124: 60–66. doi:10.1093/brain/124.1.60. PubMed: 11133787. 11133787

[21] Gousias IS, Hammers A, Counsell SJ, Srinivasan L, Rutherford MA et al. (2013) Magnetic resonance imaging of the newborn brain: automatic segmentation of brain images into 50 anatomical regions. PLOS ONE 8: e59990. doi:10.1371/journal.pone.0059990. PubMed: 23565180. 23565180 PMC3615077

[22] Maunu J, Lehtonen L, Lapinleimu H, Matomäki J, Munck P et al. (2011) Ventricular dilatation in relation to outcome at 2 years of age in very preterm infants: a prospective Finnish cohort study. Dev Med Child Neurol 53: 48–54. doi:10.1111/j.1469-8749.2010.03785.x. PubMed: 21039438. 21039438

[23] Van Kooij BJM, Hendrikse J, Benders MJNL, de Vries LS, Groenendaal F (2010) Anatomy of the circle of Willis and blood flow in the brain-feeding vasculature in prematurely born infants. Neonatology 97: 235–241. doi:10.1159/000253754. PubMed: 19887852. 19887852

[24] Van Kooij BJM, Benders MJNL, Anbeek P, Van Haastert IC, de Vries LS et al. (2012) Cerebellar volume and proton magnetic resonance spectroscopy at term, and neurodevelopment at 2 years of age in preterm infants. Dev Med Child Neurol 54: 260–266. doi:10.1111/j.1469-8749.2011.04168.x. PubMed: 22211363. 22211363

[25] Duda RO, Hart PE, Stork DG (2001) Pattern classification. John Wiley and Sons.

[26] Likar B, Viergever MA, Pernus F (2001) Retrospective correction of MR intensity inhomogeneity by information minimization. IEEE Trans Med Imaging 20: 1398–1410. doi:10.1109/42.974934. PubMed: 11811839. 11811839

[27] Klein S, Staring M, Murphy K, Viergever MA, Pluim JPW (2010) Elastix: a Toolbox for Intensity-Based Medical Image Registration. IEEE Trans Med Imaging 29: 196–205. doi:10.1109/TMI.2009.2035616. PubMed: 19923044. 19923044

[28] Anbeek P, van der Grond J, Vincken KL, Viergever MA, van Osch MJP (2005) Probabilistic Segmentation of MR Brain Images. Chapter 5: Influence of K on the Probabilistic Segmentation of MR Brain Images with K-Nearest Neighbor Classification. Utrecht University, the Netherlands.

[29] Sun Y, Wong AKC, Kamel MS (2009) Classification of Imbalanced Data: a Review. International Journal of Pattern Recognition and Artificial Intelligence 23: 687–719. doi:10.1142/S0218001409007326.

[30] Tan S (2005) Neighbor-weighted K-nearest neighbor for unbalanced text corpus. Expert Systems with Applications 28: 667–671. doi:10.1016/j.eswa.2004.12.023.

[31] Parvin H, Alizadeh H, Minaei-bidgoli B (2008) MKNN : Modified K-Nearest Neighbor. Proceedings of the World Congress on Engineering and Computer Science, WCECS. pp. 22–25.

[32] Liu W, Chawla S (2011) Class Confidence Weighted k NN Algorithms for. Advances in Knowledge Discovery and Data Mining. Lecture Notes in Computer Science: 345–356.

[33] Van Pul C, van Kooij BJM, de Vries LS, Benders MJNL, Vilanova A et al. (2012) Quantitative fiber tracking in the corpus callosum and internal capsule reveals microstructural abnormalities in preterm infants at term-equivalent age. AJNR Am J Neuroradiol 33: 678–684. doi:10.3174/ajnr.A2859. PubMed: 22194382. 22194382 PMC8050470

[34] Hagmann CF, De Vita E, Bainbridge A, Gunny R, Kapetanakis AB et al. (2009) T2 at MR imaging is an objective quantitative measure of cerebral white matter signal intensity abnormality in preterm infants at term-equivalent age. Radiology 252: 209–217. doi:10.1148/radiol.2522080589. PubMed: 19561257. 19561257

[35] Dyet LE, Counsell SJ, Maalouf EF, Ajayi-Obe M, Kennea N (2006) Natural history of brain lesions in extremely preterm infants studied with serial magnetic resonance imaging from birth and neurodevelopmental assessment. Pediatrics 118: 536–548. doi:10.1542/peds.2005-1866. PubMed: 16882805. 16882805

[36] De Bresser J (2011) MRI-based quantification of brain damage in cerebrovascular disorders. Utrecht University, The Netherlands.

[37] Bayley N (2006) Bayley Scales of Infant and Toddler Development, 3rd edition. Harcourt Assessment, San Antonio TX.

[38] Woodward LJ, Anderson PJ, Austin NC, Howard K, Inder TE (2006) Neonatal MRI to predict neurodevelopmental outcome in preterm infants. N Engl J Med, 355(7): 685-694. doi:10.1056/NEJMoa053792. PubMed: 16914704. 16914704

